# Book Reviews

**Published:** 1901-05

**Authors:** 


### *BOOK REVIEWS, PAMPHLETS, EXCHANGES.*

### *BOOK REVIEWS.*

[Contributions solicited for review.]

Nursing: Its Principles and Practice. By Isabel Adams Hampton, Graduate of the New York Training School for Nurses attached to Bellevue Hospital; Superintendent of Nurses and Principal of the Training School for Nurses, Johns Hopkins Hospital, Baltimore, Md.; Late Superintendent of Nurses, Illinois Training School for Nurses, Chicago, Illinois; Member of the Board of Lady Managers, The Lakeside Hospital, Cleveland, Ohio; Honorary Member of the Matrons' Council, London, England. In one very Handsome volume of 512 pages (size 5x7½), profusely illustrated. Price, Cloth, $2.00, net. E. Koeckert, Publisher, 702 Rose Building, Cleveland, Ohio.

This very excellent work deals with a somewhat neglected phase of the avocation of nursing—the principles and practice—the ethics of the calling. The nurse has a very important position to fill, and her equipment should include instruction in other matters than those connected with the mechanical part of her work. Such instruction may be obtained from a careful perusal of Mrs. Robb's book. Her advice and recommendations cover such ground as is concerned in the moral and educational training of the woman who essays to go forth "to aid in the practical solution of the various social problems which are mastered only by the help of intelligent womanly work."

The volume can be cordially commended, and it will doubtless become classic as its merits are appreciated.

THE MEDICAL NEWS POCKET FORMULARY. New (3d) Edition. Containing 1700 prescriptions representing the latest and most approved methods of administering remedial agents. By E. Quin Thornton, M.D., Demonstrator of Therapeutics, Pharmacy and Materia Medica in the Jefferson Medical College, Philadelphia. New (3d) edition, carefully revised to date of issue. In one wallet-shaped volume, strongly bound in leather, with pocket and pencil. Price, $1.50, net. Lea Bros. & Co., Philadelphia and New York, 1901.

This neat volume can be conveniently carried in the pocket for ready reference. It will be found "an ever-present help in time of trouble" for the young doctor who is as yet somewhat insecure on therapeutics. The subjects are arranged in alphabetical order for easy consultation. Besides this, there are tables of dosage, poisons and antidotes and incompatibles, weights and measures, and a comparative scale of the metric and apothecary system. The volume bound in red leather presents a very elegant appearance.

A COMPEND OF HUMAN PHYSIOLOGY. Especially adapted for the use of medical students. By Albert P. Brubaker, A.M., M.D., Philadelphia. Tenth Edition. P. Blakiston's Son & Co., Philadelphia, 1900. Price, 80c.

This has proven one of the most popular of the Quiz-compend series, and has been the crutch that has supported many a halting student. It presents the essential facts of the subject in a manner readily understood and learned by the medical student. The present edition has been revised and brought up to date, and can be commended as admirably filling the niche for which it is intended.

---

MEMORANDA OF POISONS. By Thomas Hawkes Tanner, M.D., F.L.S. Eighth Revised Edition. By Henry Leffmann, A.M., M.D. Philadelphia, P. Blakiston's Son & Co. 1901.

This little book is already well known and popular. It contains, besides chapters on general toxicology, a classification of the poisons, their specific effects and treatment. It will serve an excellent purpose, as it is brief and can be consulted at a moment's notice.

